# Current and Future Treatments for Takayasu Arteritis: Toward Cardiovascular Risk Modification

**DOI:** 10.1161/CIRCULATIONAHA.125.076308

**Published:** 2026-01-27

**Authors:** Alexandra Armstrong, Dan Pugh, Neil Basu, Neeraj Dhaun

**Affiliations:** Edinburgh Kidney, BHF/The Institute for Neuroscience and Cardiovascular Research, University of Edinburgh, UK (A.A., D.P., N.D.).; Institute of Infection, Immunity & Inflammation, University of Glasgow, UK (N.B.).

**Keywords:** heart disease risk factors, Takayasu arteritis, vasculitis

## Abstract

Takayasu arteritis (TAK) is a rare, immune-mediated large-vessel vasculitis that affects predominantly young women and carries a substantial risk of both vascular complications and long-term cardiovascular disease. Although glucocorticoids and conventional immunosuppressive therapies remain the cornerstone of treatment, relapse rates are high, and current strategies fail to adequately mitigate future cardiovascular risk. This review synthesizes evidence on current treatment strategies, unmet clinical needs, and novel approaches, including immunological and vascular-targeted therapies, and argues for a shift in management paradigm toward integrated cardiovascular risk reduction. We discuss advances in understanding the pathogenesis of TAK, highlighting the roles of innate and adaptive immunity in disease progression, and the challenges of early diagnosis and disease monitoring. We critically appraise current treatment paradigms, including glucocorticoids, conventional disease-modifying antirheumatic drugs, and biologics such as tocilizumab and tumor necrosis factor-α inhibitors, and outline emerging therapies targeting novel pathways, including interleukin-17, interleukin-12/23, Janus kinase/signal transducer and activator of transcription, and Notch-1/mammalian target of rapamycin complex signaling. We highlight the increasing recognition of cardiovascular morbidity as a major contributor to mortality in TAK and the need for integrated approaches to risk factor modification. We explore a road map for advancing management of cardiovascular disease in TAK, including comprehensive screening tools that integrate serological and imaging biomarkers to interrogate cardiovascular risk and potential therapeutic cardioprotective strategies such as sodium-glucose cotransporter 2 inhibitors and endothelin receptor antagonists. Despite recent progress, clinical management remains limited by diagnostic uncertainty, heterogeneous treatment approaches, and a paucity of high-quality randomized controlled trials. Future work should focus on interventions that target both immune-mediated vascular injury and cardiovascular disease progression. Achieving long-term disease remission while reducing cardiovascular mortality must become the primary therapeutic goal in TAK.

Takayasu arteritis (TAK) is a large-vessel vasculitis that affects predominantly the aorta and its main branches. It is characterized by a maladaptive immune response and chronic inflammation, which lead to intimal fibrosis, vascular narrowing, occlusions, and aneurysmal degeneration of the arteries affected. TAK affects predominantly those 20 to 40 years of age, with women making up 80% to 90% of cases.^1^ Although rare—TAK has an estimated incidence of ≈1 to 3 cases per million globally^1,2^—those affected endure a lifelong burden of disease, significantly affecting quality of life.

When active, TAK may cause a range of nonspecific symptoms, including fever, malaise, and weight loss.^3^ Clinical manifestations relate to extent of vascular involvement. Arterial narrowing may present acutely as stroke or limb ischemia or longer-term as hypertension and heart failure.^3^ Although current treatments allow patients to enter disease remission, the 10-year relapse rate is ≈50%.^4^ Mortality among patients with TAK is 2- to 3-fold greater than in control populations,^5,6^ and cardiovascular disease is the leading cause of death.^7^ Ischemic complications develop in one-third of patients at a median age of 41 years,^8^ indicating the need for more effective preventive measures. Whether the increased use of targeted therapies has improved these outcomes remains unclear.

This review is intended to orient cardiovascular clinicians and researchers to the evolving management of TAK, a disease that challenges traditional paradigms of vascular care.

We discuss current approaches to management, highlight the associated cardiovascular disease risks, and propose a road map for advancing both research and clinical practice. Particular emphasis is placed on developing strategies that address not only the immunological aspects of disease but also its cardiovascular complications.

## Disease Pathogenesis

**Figure 1.**
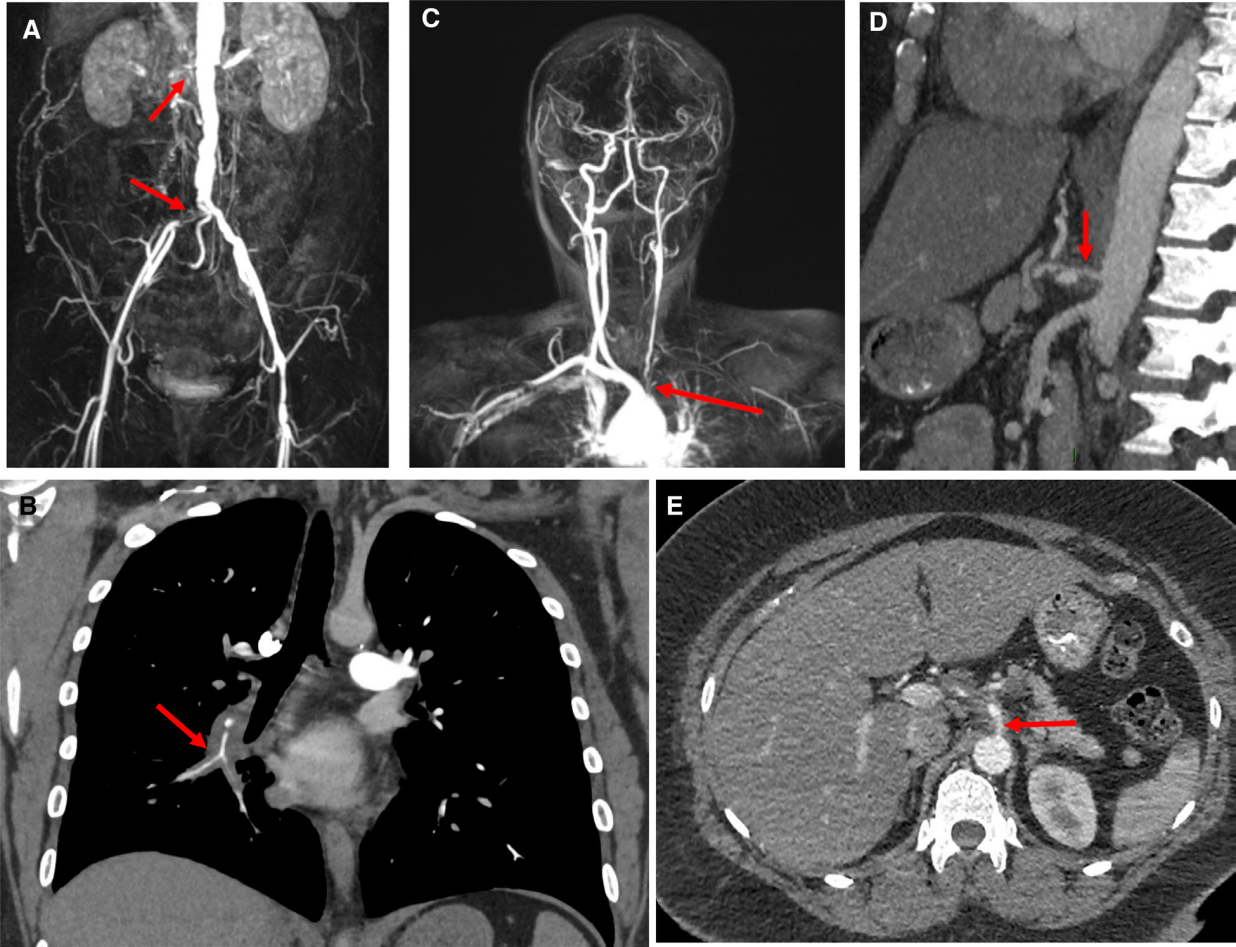
**Examples of vascular stenosis and occlusion in Takayasu arteritis. A**, Magnetic resonance angiography (MRA) showing occlusion of the right renal artery and right iliac artery. **B**, A computed tomography (CT) pulmonary angiogram showing thickening around a stenotic portion of the right lower lobe pulmonary artery. **C**, MRA showing stenosis of the left common carotid artery and proximal occlusion of the left vertebral and subclavian arteries. **D** and **E**, Same contrast-enhanced CT showing stenosis and probable dissection of the coeliac axis with surrounding inflammatory change.

TAK is characterized by immune-mediated damage to blood vessels, especially the inner arterial wall (intima). Although the pathogenesis is not fully understood, initial loss of vascular immune tolerance triggers an aberrant cascade of disease processes involving both the innate and adaptive immune systems. The disease progresses from early granulomatous inflammation to a chronic inflammatory reaction that results in smooth muscle proliferation into the vascular intima and fibrosis in the tunica adventitia with eventual vascular narrowing. The consequences of this inflammatory damage include stenosis (Figure 1), thrombosis, and aneurysm formation.

**Figure 2.**
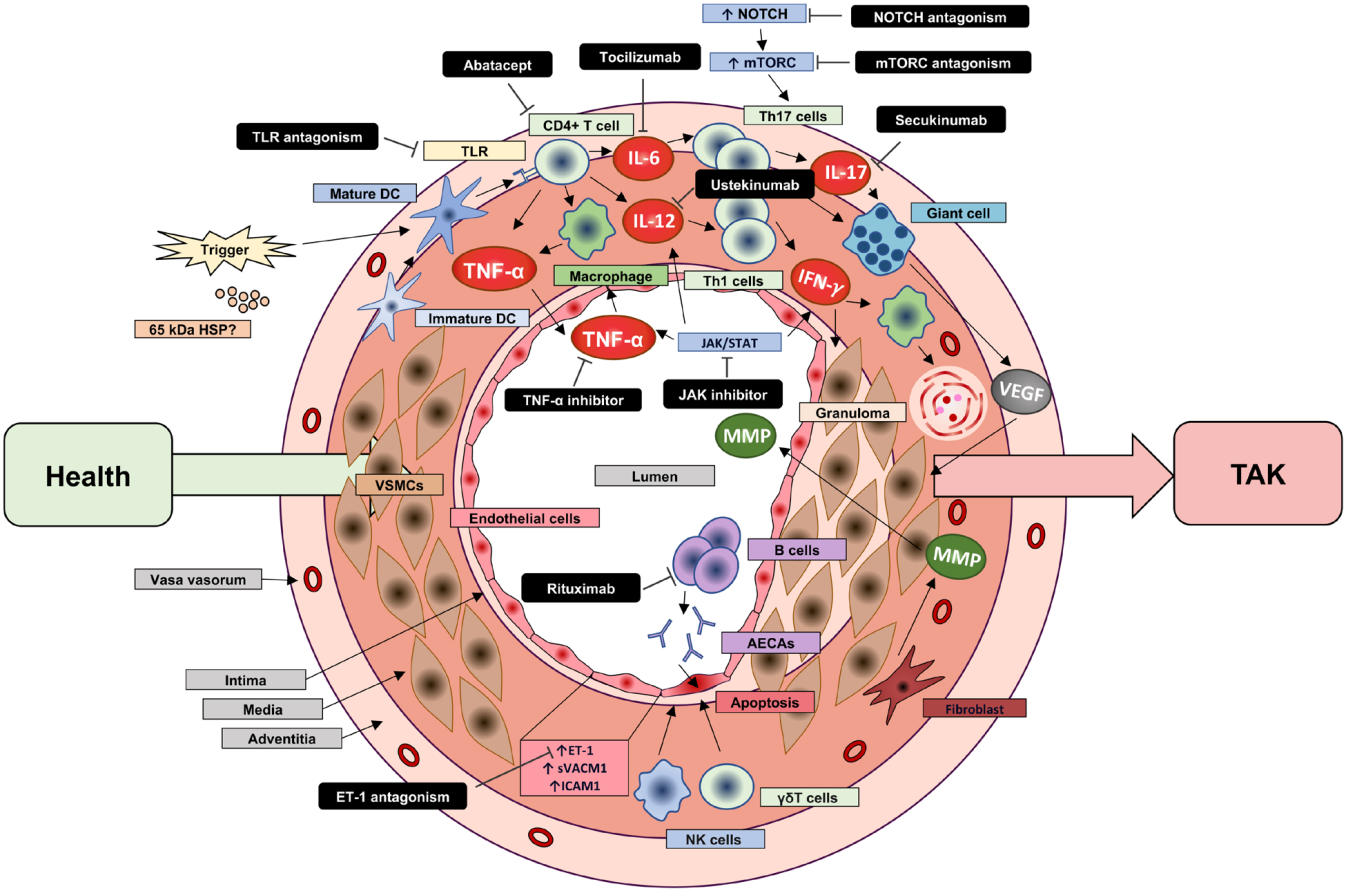
**Overview of the pathogenesis and treatments of TAK.** Takayasu arteritis (TAK) is initiated by a breakdown in immune tolerance to arterial wall antigens, potentially triggered by microbial mimics such as heat shock proteins (HSPs). Vascular dendritic cells orchestrate the recruitment and activation of CD4⁺ T cells through Toll-like receptors (TLRs). Monocyte activation and dysregulated NOTCH/mammalian target of rapamycin complex 1 (mTORC1) and Janus kinase (JAK)/STAT signaling recruit T helper (Th) 1 and Th17 responses. This results in secretion of proinflammatory cytokines, including interferon-γ (IFN-γ) and tumor necrosis factor-α (TNFα). These mediators promote monocyte activation, granuloma formation, and chronic inflammation. Macrophage-derived factors, including vascular endothelial growth factor (VEGF) and matrix metalloproteinases (MMPs), drive neovascularization, adventitial fibrosis, and intimal hyperplasia. Concomitantly, activated B cells produce anti-endothelial autoantibodies (AECAs), facilitating endothelial cell apoptosis through both antibody-dependent and direct cytotoxic mechanisms. Collectively, these immune processes culminate in chronic vessel wall inflammation, remodeling, and luminal stenosis. Many of these pathways are being targeted therapeutically in current and future treatments. DC indicates dendritic cell; ET-1, endothelin-1; ICAM1, intracellular adhesion molecule 1; IL, interleukin; NK, natural killer; sVCAM1, soluble vascular cell adhesion molecule 1; and VSMC, vascular smooth muscle cell.

Although disease triggers are yet to be elucidated and our understanding of the immune mechanisms remains far from definitive, many pathways and effectors have been implicated in disease progression (Figure 2). Although this complex network of mechanisms offers a broad range of potential therapeutic opportunities, it also poses significant clinical challenges in establishing a definitive treatment pathway.

## Diagnosis and Management of TAK

Because the early clinical manifestations of TAK are heterogeneous, there is often diagnostic delay. One study reported an average of 17.5 months from clinical presentation to definitive diagnosis.^6^ Consequently, established vascular damage may be present from the outset, with progressive aortic pathology, relapsing hypertension, and atherosclerosis contributing to morbidity. Currently, a diagnosis of TAK relies on a multifaceted approach incorporating symptoms, blood tests, and imaging.

The most widely adopted classification criteria are traditionally the modified Ishikawa criteria^9^ and more recently the 2022 American College of Rheumatology/ European Alliance of Associations for Rheumatology (EULAR) criteria.^10^ These combine characteristic symptoms and clinical signs such as limb claudication, diminished pulses, and discrepancies in blood pressure between limbs with laboratory measures and vascular imaging abnormalities. Because clinical manifestations are more evident in the later stages of disease, vascular imaging has a critical role in disease diagnosis.

The optimal imaging modality for TAK diagnosis (and monitoring) remains undefined. Options include ultrasonography, computed tomography, magnetic resonance imaging (MRI), and ^18^F-fluorodeoxyglucose positron emission tomography (PET). Although both computed tomography and MRI visualize vascular lesions and complications clearly, MRI is often preferred because of the lower radiation dose.^11^ ^18^F-fluorodeoxyglucose PET defines areas with increased metabolic activity, and its combination with computed tomography or MRI allows more precise anatomic localization of inflammation. However, the use of these modalities is limited by access to scanners, expense, and suitably qualified operators.^11^

Once a diagnosis of TAK is secured, distinguishing between active and quiescent disease remains a clinical challenge. Remission is defined as the absence of new symptoms, a lack of raised inflammatory markers, and a lack of disease progression on imaging.^12^ There remain no gold standard investigations to monitor disease activity. Established laboratory biomarkers are unreliable in this context.^13,14^ In a study of patients with large-vessel vasculitis, including 56 patients with TAK, erythrocyte sedimentation rate did not correlate with patient-reported outcomes or PET imaging, and C-reactive protein demonstrated only a modest correlation.^15^

Although several novel biomarkers of disease activity have been proposed (eg, matrix metalloproteinase-3,^16^ metalloproteinase-9,^16^ pentraxin 3,^14^ and interleukin [IL]-6^17^), none have transitioned successfully into clinical practice. The Disease Extent Index.Takayasu was developed to monitor disease activity according to clinical findings alone. However, 1 study found that 14% of patients with a Disease Extent Index.Takayasu score of 0 had active disease according to physician assessment.^18^ Imaging modalities have been trialed in disease monitoring, with a recent study examining the utility of ^18^F-fluorodeoxyglucose PET/MRI for longitudinal disease assessment in large-vessel vasculitis.^19^ The study recruited 24 patients, including 6 with TAK, and demonstrated that PET and MRI metrics were able to distinguish active from inactive disease and to detect changes in activity over time.

The challenges with diagnosis and disease monitoring make evaluating new treatments for TAK difficult. Diagnostic doubt leads to many patients being excluded from trials that already struggle to recruit enough patients. In addition, given the complexities in distinguishing relapse and remission and the lack of gold standard for disease monitoring, it is difficult to define reliable study end points. These factors, along with its orphan disease status, all contribute to pharma hesitancy in undertaking clinical trials in this space. Consequently, few randomized controlled trials (RCTs) exist, limiting the evidence available for drug efficacy and comparisons between treatments. Current evidence stems mainly from observational and open-label studies, making therapeutic decisions difficult for clinicians and contributing to morbidity. Accordingly, individualized approaches are common, and significant treatment heterogeneity exists.

## Current Treatments

The management of TAK involves 2 main stages: induction of disease remission and remission maintenance. The primary goal of remission induction is to suppress vascular inflammation. Current recommendations suggest a combination of glucocorticoids and a glucocorticoid-sparing agent from the outset.^20,21^ Potential glucocorticoid-sparing agents include conventional disease-modifying antirheumatic drugs (cDMARDs) such as methotrexate or azathioprine or biologic agents such as tumor necrosis factor-α (TNFα) inhibitors or the IL-6 receptor antagonist tocilizumab.

**Figure 3.**
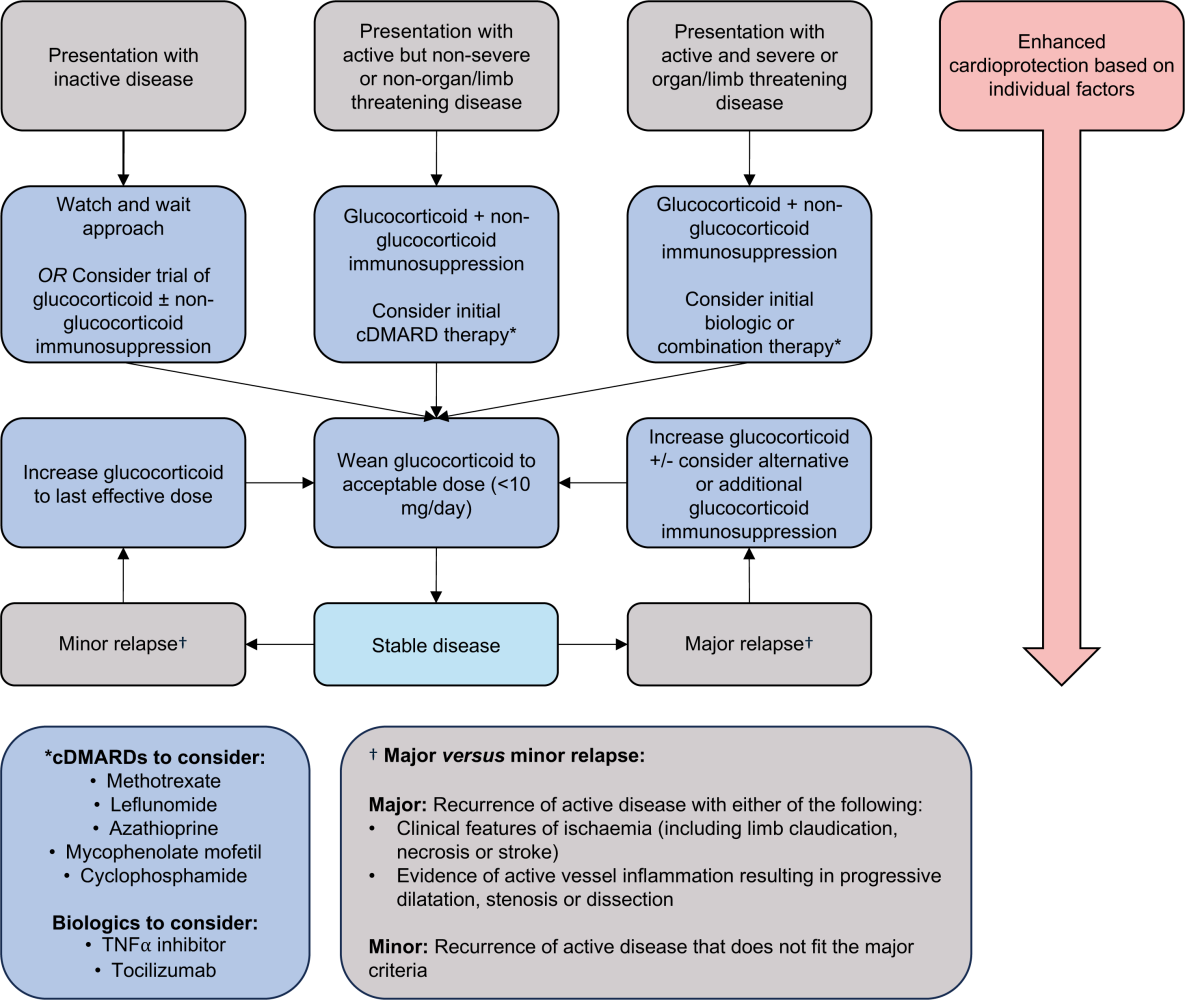
**Management of TAK.** Schematic outlining a simplified approach to the management of Takayasu arteritis (TAK). Glucocorticoid and a glucocorticoid-sparing agent is recommended from the outset of diagnosis of active TAK. Choice of glucocorticoid-sparing agent is determined by physician preference and disease severity. Addition of cardioprotective medications is based on an individual’s risk of developing cardiovascular disease. cDMARD indicates conventional disease-modifying antirheumatic drug; and TNFα, tumor necrosis factor-α.

Once initial disease control is achieved, glucocorticoids are gradually tapered to minimize adverse effects. At this stage, relapse is not uncommon, necessitating substitution of the glucocorticoid-sparing agent or re-escalation of glucocorticoid dose. The available guidance for choice of glucocorticoid-sparing agent is based on relatively low-quality evidence given the absence of RCTs, with a treatment framework algorithm outlined in Figure 3. In practice, the choice of glucocorticoid-sparing drug often comes down to physician preference and experience. Regular imaging follow-up is essential to monitor disease activity, to assess treatment response, and to inform adjustments to therapeutic strategy.

### Glucocorticoids

Both the American College of Rheumatology and EULAR recommendations suggest initiating high-dose glucocorticoid in active TAK.^20,21^ The initial recommended dose varies depending on perceived disease activity and severity and the potential for adverse effects. Evidence for glucocorticoid monotherapy is limited, and relapse rates of ≈60% have been reported during tapering in the control arms of the tocilizumab and abatacept clinical trials.^22,23^ Long-term high-dose glucocorticoid treatment is impractical given its adverse effects: fractures, glaucoma, and increased risk of diabetes, infections, osteoporosis, and death. Tapering usually starts after 2 to 4 weeks, once disease control is achieved. EULAR guidelines propose a target dose of ≤10 mg/d within 1 year,^20^ a dose considered high given the increased risk of cardiovascular events associated with doses as low as <5 mg.^24^ In practice, maintenance glucocorticoid dose is dictated by individual disease and patient characteristics.

### Conventional Immunosuppression

Because access and cost of biologic disease-modifying antirheumatic drugs limit their use, cDMARDs have traditionally been used as first-line adjunctive therapy to glucocorticoid, although evidence is limited to small studies and retrospective data. Methotrexate is the most common choice, with open-label studies reporting clinical improvement in >75% of patients when combined with glucocorticoid.^25^ However, nearly half of patients experienced a relapse as glucocorticoid was tapered and required retreatment. Leflunomide has demonstrated comparable efficacy to methotrexate in retrospective comparisons.^26^ Longer-term follow-up, however, suggests a high risk of relapse or treatment discontinuation.^27^ Its role will be clarified by the ongoing TACTIC trial (Takayasu Arteritis Clinical Trial in China) comparing leflunomide with placebo.^28^

Azathioprine combined with glucocorticoid has shown efficacy in small cohorts,^29^ and retrospective studies indicate similar relapse and adverse event rates between azathioprine and methotrexate.^30^ In the same cohort, it was less effective than methotrexate in reducing glucocorticoid use and preventing surgical intervention. Evidence for mycophenolate mofetil is limited but indicates potential benefit in reducing inflammatory markers, reducing glucocorticoid dose and improving disease activity.^31^ Cyclophosphamide reduced disease activity and improved imaging findings compared with methotrexate,^32^ but its use is restricted in TAK because of fertility concerns in young women, despite proven efficacy in other vasculitides.^33^

### Biologic Disease-Modifying Antirheumatic Drugs

Although previously reserved for when cDMARDs fail, biologics are increasingly used as first-line treatments for TAK. Unlike cDMARDs, which have broad immunosuppressive effects, biologic disease-modifying antirheumatic drugs target specific pathways in the immune response.

#### Tocilizumab

Tocilizumab is a monoclonal antibody that is approved for several rheumatic diseases and has shown promise in TAK. It selectively binds to IL-6, blocking interaction with its cognate receptor and preventing a cascade of proinflammatory effects.^22^ Patients with TAK have elevated circulating IL-6 concentrations, which correlate with disease activity, underscoring the potential of tocilizumab as a therapeutic option in this space.^34^ In the TAKT study (Takayasu Arteritis Treated With Tocilizumab), a double-blind phase 3 RCT, there was a favorable trend toward tocilizumab over placebo for time to relapse, the primary end point; the relapse rate was higher in the placebo arm (53% versus 23%).^22^ In the longer open-label follow-up in which all patients received tocilizumab for 96 weeks, disease stabilization was observed in 70% and improvement was seen in 20%.^35^ One challenge is that routinely measured acute-phase reactants become unreliable indicators of disease flare in patients receiving tocilizumab. Data from the GiACTA trial (Giant-Cell Arteritis Actemra), a randomized, placebo-controlled phase 3 trial of tocilizumab in giant-cell arteritis, concluded that C-reactive protein levels were suppressed in nearly all patients treated with tocilizumab, including those with disease flares.^36^ Given the increasing use of tocilizumab in TAK, new biomarkers that reliably define disease activity are urgently needed.

#### TNFα Inhibitors

TNFα is key to the development of granulomatous inflammation in TAK. Serum TNFα concentrations are higher in patients with TAK compared with controls,^37^ and T-cell production of TNFα is higher in active TAK compared with remission.^38^ TNFα inhibitors, including adalimumab and infliximab, are widely used in rheumatic diseases, and evidence supports their use in TAK. A meta-analysis of 19 observational studies showed partial or complete clinical response in 80% of patients and angiographic stabilization in 86% in the studies that assessed these outcomes.^39^ Treatment with TNFα inhibitors also leads to improvements in quality of life measures such as perceived body pain.^40^

Comparative data suggest that TNFα inhibition may be more effective than conventional therapies. A Norwegian observational study reported only 10% of patients treated with TNFα inhibitors developed new vascular lesions over 2 years compared with 40% of those receiving cDMARD and 92% on glucocorticoid monotherapy.^41^ Remission rates were also higher with TNFα inhibition than with cDMARDs (42% versus 20%). The American College of Rheumatology recommends TNFα inhibition over tocilizumab for the treatment of refractory TAK,^21^ partly because of the inconclusive TAKT study. EULAR guidelines state that either agent may be used.^20^ Meta-analyses and retrospective studies have not demonstrated a clear difference in remission or safety between the 2 approaches.^42^ It should be recognized that different centers approach TAK differently, so comparator studies may be biased by patients with difficult-to-treat disease being prescribed tocilizumab or TNFα inhibitors after failure of first-line management. INTOReTAK (Infliximab and Tocilizumab in Refractory/Relapsing Takayasu Arteritis; NCT04564001) is currently recruiting and will assess the efficacy and safety of infliximab compared with tocilizumab in TAK.

#### Abatacept

Abatacept, which inhibits T-cell activation by acting as a negative regulator of CD28 costimulation,^23^ has not demonstrated benefit in TAK, despite reports of increased expression of T-cell costimulatory molecules in inflamed vasculature in TAK. In a phase 2 trial, it failed to prolong remission compared with placebo (5.5 months versus 5.7 months).^23^ Notably, relapse-free survival at 12 months was 22% for abatacept-treated patients and 40% for those receiving placebo. Although the study cited the small sample size as a limitation, neither the American College of Rheumatology nor EULAR includes abatacept in their list of recommended treatments for TAK.^20,21^

## Emerging Treatments

Current therapies for TAK have limitations. Both disease relapse risk (≈50%)^4^ and mortality rate (≈10%) at 10 years are high.^43^ Thus, more effective treatments that target the complex pathophysiology of TAK and its longer-term sequelae are needed. A range of emerging therapies have been proposed and are summarized in the Table.

**Table 1.**
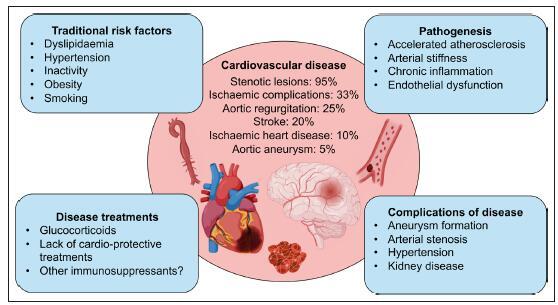
Summary of Evidence for Emerging Immunosuppressant Therapies in TAK

## Combination Treatment

An increasingly adopted strategy to improve treatment efficacy is the use of combination immunosuppressive therapy. This approach is well established in other inflammatory conditions, including rheumatoid arthritis and systemic erythematosus. In TAK, an RCT demonstrated that mycophenolate mofetil with methotrexate achieved a significantly higher complete response rate at 1 year compared with cyclophosphamide followed by azathioprine (55% versus 32%).^63^ The multicenter trial included 111 patients, achieving a relatively large sample size. These findings highlight combination therapy as a promising treatment strategy that may offer superior efficacy compared with a single nonglucocorticoid treatment regimen.

## Cardiovascular Disease Risk

**Figure 4.**
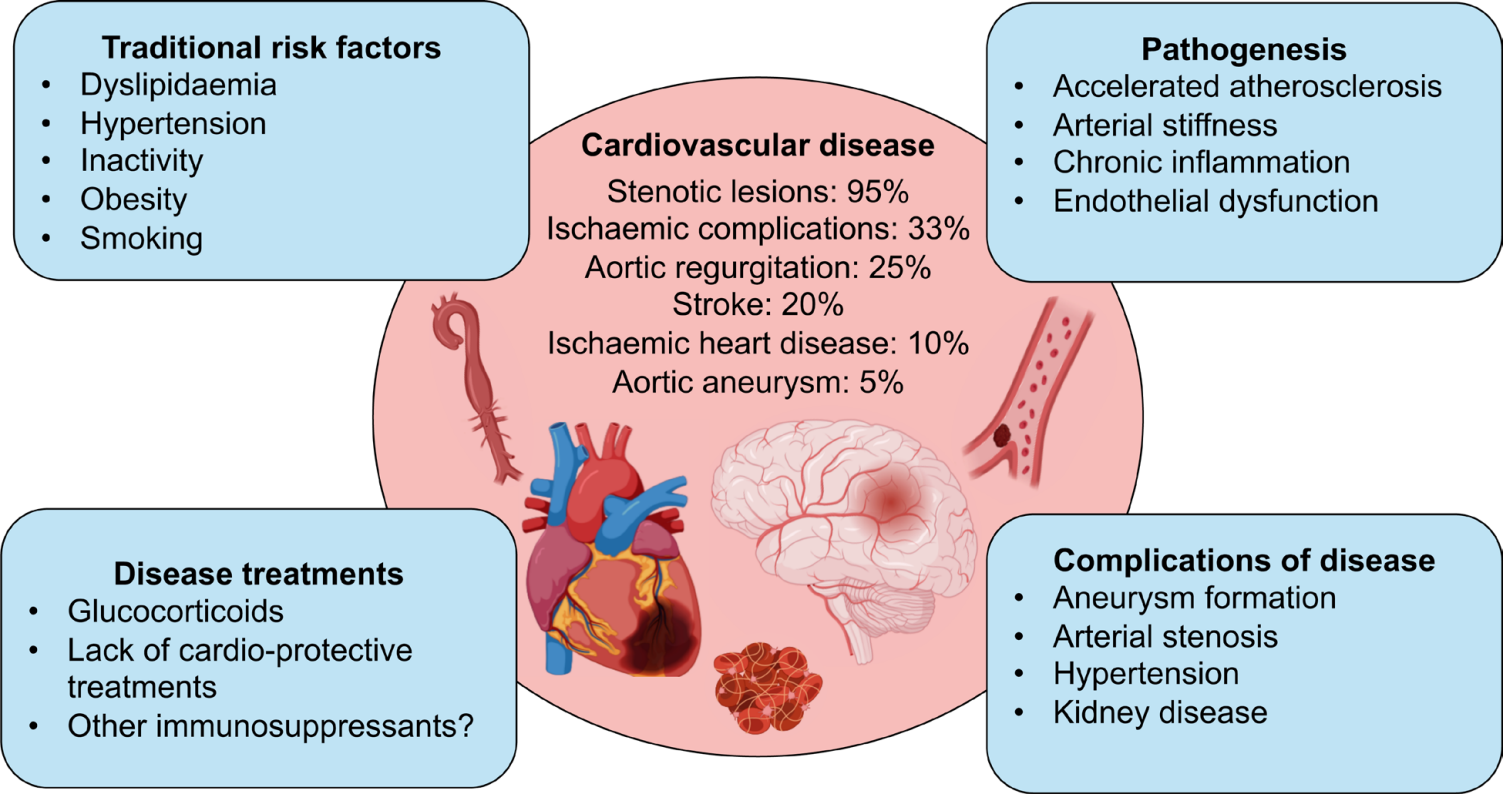
**Factors influencing cardiovascular disease development in TAK.** Schematic outlining the factors influencing cardiovascular disease development and subsequent prevalence of cardiovascular events in Takayasu arteritis (TAK). Cardiovascular disease includes stenotic lesions,^65,66^ ischemic complications,^8^ aortic regurgitation,^67^ stroke,^68^ ischemic heart disease,^5^ and aortic aneurysm.^66^

TAK is associated with substantial morbidity and mortality. Cardiovascular disease is the leading cause of mortality,^7,64^ with >80% of deaths directly attributable to cardiovascular complications,^7^ although there is a need for updated longitudinal data to determine whether treatment advances have improved long-term outcomes. Cardiovascular complications can be attributed both to the immediate effects of active disease, arterial inflammation, destruction, and stenosis and to the accrual of longer-term cardiovascular dysfunction, which can be multifactorial (Figure 4).

Across the vascular system, lesions attributable to active TAK are common, with an average of 2.6 lesions per patient (range, 1–10).^69^ These need to be monitored, and lesions posing a high complication risk may require revascularization, with surgical intervention rates ranging from 40% to 75% across different cohorts.^65,70^ Despite these measures, complications still arise; ischemic events are common and occur at a relatively young age. In 1 cohort with a median age of 37.5 years, 33% of patients had already experienced an ischemic complication.^8^ Cerebrovascular ischemic events are particularly frequent, affecting ≈15% to 20% of patients, with lasting neurological sequelae.^68,71^

Other cardiovascular manifestations are also prevalent. Coronary artery involvement has been reported in 5% to 60% of patients across different cohorts.^72^ One study reported that coronary events (including angina and myocardial infarction) occurred in 11% of patients over a 5.6-year follow-up.^73^ Data from coronary computed tomography angiography have demonstrated coronary lesions in half of patients; only 30% had cardiac symptoms, suggesting that in clinical practice the prevalence of the lesions is underestimated.^74^ Factors associated with the development of coronary lesions include older age at disease onset, longer disease duration, and the presence of traditional risk factors.^75^ Although revascularization therapy is currently an option for intervention, the optimal management strategy for coronary artery lesions remains to be fully established.

In addition to coronary lesions, other cardiovascular manifestations are also prevalent, with estimates ranging from 40% to 60% of patients.^76,77^ These may arise from direct coronary artery inflammation or extension from aortitis. Cardiac structures that can be involved include the pericardium, myocardium, and heart valves. Clinical manifestations include aortic valve insufficiency, myocarditis, pericarditis, and intracardiac thrombus formation. Valvular regurgitation is common; in a cohort of 1069 patients, 35% had valvular regurgitation, most frequently aortic regurgitation (70%), with nearly half graded as moderate to severe.^67^ Aortic aneurysms occur in ≈4% of patients and are typically attributable to TAK aortic disease.

### Mechanisms of Cardiovascular Disease

Mechanisms explaining the increased longer-term cardiovascular risk in TAK remain poorly understood. In addition to direct disease effects such as arterial inflammation and damage, contributing factors likely include traditional risk factors, accelerated atherosclerosis, and the impact of disease-modifying therapies (Figure 4). Traditional risk factors are more prevalent in TAK,^78^ and patients tend to demonstrate reduced aerobic capacity, reduced lower-limb muscle strength, and increased visceral adipose tissue.^79^ Smoking correlates with worsening symptoms, disease progression, and the need for revascularization.^80^

#### Dyslipidemia

A high prevalence of dyslipidemia has been reported in untreated patients with TAK compared with the general population.^81^ Serum lipid dysregulation was shown to correlate with disease activity, suggesting that systemic inflammation may play a mechanistic role. Proinflammatory cytokines central to the pathogenesis of TAK, including TNFα and IL-6, are known to alter lipid metabolism and to lead to an atherogenic lipid profile.^82^ This is consistent with observations in other inflammatory diseases such as psoriasis, in which dyslipidemia is a common complication.^82^ Hyperlipidemia was evident in untreated patients,^81^ highlighting that this occurs even before the addition of glucocorticoids, which are known to promote dyslipidemia.^83^ The presence of dyslipidemia is clinically relevant in TAK; patients who experience ischemic events more frequently have hyperlipidemia.^8^ These findings underscore the importance of early identification and management of dyslipidemia in this population.

#### Hypertension

Hypertension occurs in ≈70% to 80% of patients with TAK,^79,84^ with development often related to decreased vascular compliance or renal artery stenosis. It frequently presents early in the disease course, with 1 study reporting a mean age at onset of 25 years, with ≈30% of patients developing hypertension before 20 years of age.^85^ Blood pressure should be routinely checked in all 4 limbs and managed aggressively. No class of antihypertensive agents has demonstrated superiority in TAK, although comparative head-to-head trials are lacking. Reflecting this, current guidance does not provide specific recommendations.^20,21^ How physicians manage and monitor blood pressure in TAK is not well established; however, 1 study reported that only 40% of patients were prescribed antihypertensive drugs, with 33% prescribed an angiotensin-converting enzyme inhibitor and 22% prescribed a β-blocker.^8^

#### Disease-Modifying Treatments

Glucocorticoids, although effective in suppressing inflammation, are associated with adverse cardiovascular events. Their use promotes hypertension, dyslipidemia, hyperglycemia, and increased central obesity.^83^ Doses as low as 5 mg daily double the risk of cardiovascular disease, and higher doses (>25 mg) increase the risk 6-fold in patients with inflammatory diseases.^24^ This is concerning in TAK, with patients often requiring prolonged glucocorticoid use, and EULAR guidelines propose a target glucocorticoid dose of ≤10 mg/d within 1 year.^20^

The impact of nonglucocorticoid immunosuppression on long-term cardiovascular health in TAK remains less clear. Tocilizumab has been shown to increase low-density lipoprotein cholesterol in rheumatoid arthritis,^86,87^ potentially as a result of reduced hepatic low-density lipoprotein receptor expression. Whether these effects translate to an increase in cardiovascular risk is unclear. The risk of TNFα inhibitors is also debated, with studies displaying both cardioprotective and harmful associations.^88^ One study found that in patients with axial spondylarthritis, TNFα inhibition associated with reduced cardiovascular risk,^89^ although this association was weakened when concentrations of inflammatory markers were considered. The ORAL Surveillance trial (Safety Study of Tofacitinib Versus Tumor Necrosis Factor [TNF] Inhibitor in Subjects With Rheumatoid Arthritis) reported a higher incidence of major cardiovascular events with the use of the JAK inhibitor tofacitinib use compared with TNFα inhibitors in rheumatoid arthritis,^90^ promoting regulatory caution. However, subsequent studies have produced conflicting results,^91^ and clarification is needed.

#### Accelerated Atherosclerosis

TAK and atherosclerosis are distinct pathological conditions; however, they share similarities and in late phases can be difficult to differentiate. An increased burden of atherosclerotic lesions in patients with TAK has been demonstrated in ultrasonography studies,^92^ with 1 study reporting carotid plaques to be ≈10 times more frequent in patients with TAK than in matched controls, even after adjustment for traditional atherosclerosis risk factors.^93^

Multiple mechanisms are likely to contribute to this accelerated atherosclerotic process, including long-standing vascular inflammation with immune cell infiltration, endothelial dysfunction, dyslipidemia, and structural vascular remodeling with increased arterial stiffness. Local calcification in inflamed arterial segments has also been observed, further supporting the role of site-specific vascular injury.^94^ In addition, prolonged disease duration and long-term glucocorticoid use may amplify cardiovascular risk through adverse effects on lipid metabolism, blood pressure, and glucose homeostasis. Early initiation of statin therapy may help prevent or stabilize plaques in patients with TAK.

#### Endothelial Dysfunction and Arterial Stiffness

The endothelium is an important regulator of vascular tone and maintains vascular health, and endothelial dysfunction promotes vascular remodeling and intimal hyperplasia and consequently arterial stiffness and hypertension. In TAK, flow-mediated dilation, a measure of endothelial function, was found to be significantly reduced in a study of 32 patients.^95^ In addition, carotid intima-media thickness was increased in both active and inactive phases of the disease, further indicating persistent endothelial dysfunction and arterial stiffness. Supporting this, a meta-analysis of 3 studies demonstrated impaired arterial stiffness in TAK, measured by the gold standard carotid-femoral pulse wave velocity.^96^ No studies have yet examined the direct relationship between pulse wave velocity or flow-mediated dilation and cardiovascular event rates in TAK.

### Current Approaches to Cardiovascular Protection

Although patients with TAK face an increased long-term risk of cardiovascular disease, cardioprotective strategies remain underused. Despite evidence suggesting potential benefits, statin use in TAK remains limited. One study reported that only 32% of patients were prescribed statins, with 27% already taking them at the time of diagnosis.^8^ In a cohort of 74 patients with TAK, those on statins were less likely to relapse.^97^ This was attributed to the pleiotropic effect of statins given that it appeared to be independent of the cholesterol lowering. However, findings are not consistent; another study found no association between statin use and rates of ischemic events.^98^ Interpretation of these results is limited by the small sample sizes inherent to studies of rare disease. There is an urgent need for adequately powered trials of cardioprotective drugs in TAK. In the interim, given the prevalence of accelerated atherosclerosis in TAK, statin therapy should be considered for all patients.

Antiplatelet therapy has also been investigated for its potential cardioprotective role in TAK. One study reported that both low- and high-dose aspirin is safe and protective against acute ischemic events in patients with TAK.^98^ EULAR guidelines recommend consideration of antiplatelet therapy after individual evaluation, taking into account cardiovascular risk factors and the extent of vessel stenosis. Other studies have found no significant association between aspirin use and vascular complication–free survival,^8^ a finding that may be confounded by the fact that patients with more aggressive disease are more likely to receive aspirin. Anticoagulant therapy has not been shown to reduce ischemic events,^98^ although the low prescribing rate (≈10%)^8,98^ limits any meaningful conclusions.

The cardioprotective benefits of antihypertensive agents have not been interrogated in TAK, and no data exist on their association with relapse rates or vascular complications. Despite the high prevalence of hypertension in this patient population, studies have reported prescribing rates for any antihypertensive agent as low as 40%,^8^ highlighting a significant gap in optimal blood pressure management.

### Newer Approaches to Cardiovascular Protection

**Figure 5.**
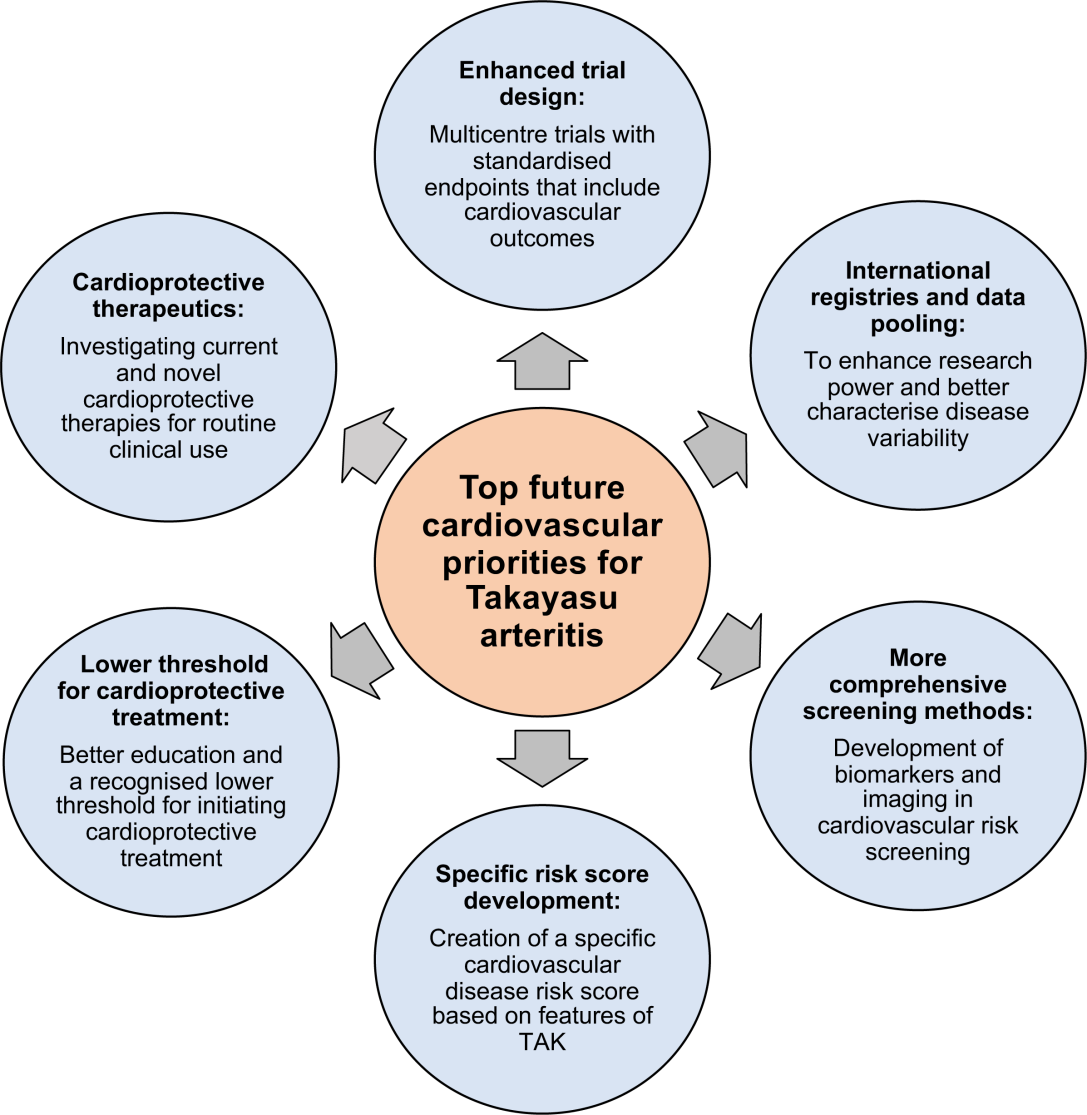
**Future cardiovascular disease priorities in TAK.** TAK indicates Takayasu arteritis.

Several potential strategies exist to reduce cardiovascular risk in patients with TAK, encompassing both screening and optimized management. These are summarized in Figure 5. Although these strategies remain central to improving outcomes, it is equally important to assess the extent to which both patients and physicians recognize the elevated cardiovascular risk associated with TAK. The TAK-IMPACT (Investigating the Management, Perspectives, and Attitudes Towards Care in Takayasu Arteritis) questionnaire, designed for physicians and patients with TAK, will provide valuable insights into current awareness and attitudes toward cardiovascular disease in this population.

#### Identifying Those at Increased Risk

Identifying patients with TAK who may be at an increased risk of developing cardiovascular complications would be helpful to allow stratified management plans. Existing tools that predict cardiovascular events in the general population underestimate risk in inflammatory conditions. For example, a doubling of the Framingham risk score improves the accuracy in systemic lupus erythematosus,^99^ whereas in rheumatoid arthritis, guidelines suggest multiplying scores by 1.5 unless rheumatoid arthritis is already incorporated as a risk factor.^100^ In TAK, no recommended adjustment factor exists, although is likely to be warranted. The development of a TAK-specific risk score such as the PREDICTS (Predictors of Risk for Elevated Flares, Damage Progression, and Increased Cardiovascular Disease in Patients With SLE) score in systemic lupus erythematosus^101^ would be useful, although the rarity of the condition would present a challenge and would require international data pooling.

Cardiovascular imaging may also have predictive value. Current imaging strategies in TAK are designed primarily to assess vascular inflammation and structural damage, both of which clearly affect risk of future events. In addition, however, basic cardiovascular investigations such as ECG and echocardiogram are rarely deployed in TAK despite being quick, inexpensive, and widely available. Other modalities such as cardiac magnetic resonance imaging may further contribute to the global assessment of cardiovascular disease^102^ but have limitations related to accessibility, cost, and patient tolerability. Last, PET imaging may hold particular value. PET imaging, traditionally with fluorodeoxyglucose radiotracer, is often deployed for disease activity assessment in TAK but may also provide information on atherosclerotic burden. Novel radiotracers, in particular the somatostatin receptor 2 ligand ^68^Ga-DOTATATE, have shown value here.^103,104^ Combining this imaging modality for the dual purpose of disease activity assessment and cardiovascular risk prediction is an exciting premise.

Serological biomarker studies in TAK have also focused primarily on inflammation and disease activity. Biomarkers that can predict cardiovascular risk in TAK are lacking and need to be identified, as they have been in other inflammatory conditions. Several biomarkers in rheumatoid arthritis are associated with cardiovascular events such as vascular cell adhesion molecule, endothelial growth factor, IL-6, matrix metalloproteinase-1, serum amyloid, and osteoprotegerin.^105,106^ Translation of these findings to TAK is plausible given the overlapping mechanisms of systemic inflammation, endothelial dysfunction, and vascular remodeling. Future research should focus on evaluating these candidate biomarkers and others in TAK to identify predictors of cardiovascular outcomes that could guide risk stratification and cardiovascular management. However, given the rarity of TAK, adequately powered studies will likely require international collaboration and pooled patient cohorts.

#### Management

Given the increased prevalence of accelerated atherosclerosis and cardiovascular morbidity in patients with TAK, prophylactic cardioprotective treatment may be used in patients with a seemingly low cardiovascular disease risk. Statins and blood pressure control should be initiated at a lower risk threshold than typically recommended in standard clinical practice.

In addition, a range of therapeutics have shown promise across a range of conditions and may be of benefit in TAK. Inhibitors of the sodium-glucose cotransporter 2 target the renal proximal tubule to promote glycosuria. Large studies have shown their impressive cardiovascular benefits across chronic kidney disease, diabetes, and heart failure.^107–109^ Although not yet studied in TAK, the sodium-glucose cotransporter 2 inhibitor canagliflozin has been shown to suppress T-cell activation in systemic lupus erythematosus and rheumatoid arthritis by inhibiting Myc- and mTORC1-driven pathways, key regulators of T-cell metabolism and growth.^110^ Its additional cardioprotective effects support a potential role for sodium-glucose cotransporter 2 inhibitors in TAK.

Glucagon-like peptide 1 receptor agonists, first developed for diabetes, regulate appetite, promote insulin secretion, and inhibit glucagon release. These agents impressively reduce cardiovascular risk in both patients with and those without type 2 diabetes.^111,112^ A population-based study has demonstrated a reduction in cardiovascular risk and death in those with immune-mediated inflammatory disease and concomitant diabetes,^113^ and trials in progress aim to determine their benefits in heart failure and peripheral arterial disease.^114^ A few data suggest a direct anti-inflammatory effect of glucagon-like peptide 1 receptor agonists in pathways relevant to TAK, with reductions of TNFα and IL-6.^115^ Whether the same benefits might be expected in TAK and in those without diabetes remains to be seen.

Recent research in the context of kidney disease has focused on targeting the endothelin system. Endothelin-1 (ET-1) is the most potent endogenous vasoconstrictor. ET-1 contributes to endothelial dysfunction, a feature of TAK,^95^ by promoting migration of vascular smooth muscle cells into the intima, leading to vascular remodeling and intimal hyperplasia.^116^ Plasma ET-1 is elevated in patients with TAK compared with controls in both active and inactive disease.^78^ ET-1 receptor antagonists are licensed for pulmonary arterial hypertension and in combination with renin-angiotensin-aldosterone-system blockers for chronic kidney disease. In small-vessel vasculitis, blockade of the ET-1 system improved arterial stiffness and endothelial function, both independent cardiovascular risk factors.^117^ To date, no study has evaluated an ET-1–blocking approach in TAK, but this has the potential to reduce vascular remodeling and to improve cardiovascular outcomes for these patients.

## Surgical Management

Revascularization procedures may be considered in the management of TAK, particularly for patients with significant vascular stenosis, occlusion, or lesions that pose a high risk of complications such as organ ischemia. Guidelines recommend that these procedures ideally be performed when disease is in remission to reduce complications.^20^ However, there is a lack of evidence for the optimal revascularization procedure for vascular lesions. Data indicate a higher durability^118^ and a lower rate of restenosis^70^ after open surgical repair compared with an endovascular approach. One study reported an overall success rate of 79% for open surgery compared with 52% for angioplasty after a median follow-up of 6 years.^65^ However, other studies report more comparable success rates,^119^ with serious complications such as bleeding and death being more common in the surgery group.^70^

Current endovascular methods include balloon angioplasty, cutting balloon angioplasty, and stenting; surgical approaches include primarily bypass operations. Balloon angioplasty might be preferred to stenting because of evidence suggesting better long-term outcomes for treating TAK-related renal artery stenosis.^120^ Repeating failed endovascular procedures could improve success rates in managing vascular complications in TAK. One study demonstrated that by repeating procedures, up to 5 times in some cases, success rates increased from 49% to 83%.^69^ Last, advances in new angioplasty techniques such as sirolimus-coated stents, which inhibit endothelial proliferation and reduce the chance of restenosis, might be integrated into future practice.^121^ Given the rarity of the condition, success with surgical or endovascular interventions may be limited by lack of experience. In this setting, regular multidisciplinary working and a tertiary referral model may improve outcomes.

## Beyond Cardiovascular Disease Risk

Although reducing cardiovascular morbidity and mortality remains a key therapeutic goal in TAK, noncardiovascular manifestations also have a major impact on patients’ lives. Symptoms such as fatigue, chronic claudication and lightheadedness can lead to significant functional limitations, disability, and psychosocial burden.^122^ Patients may experience and prioritize these disease-specific symptoms differently from clinicians, highlighting the need for a patient-centered approach to management. Furthermore, clinical trials should routinely include patient-reported outcomes to ensure that therapies address disease activity, cardiovascular disease, and quality-of-life considerations.

## Conclusions

Despite significant progress in understanding and managing TAK, optimal care remains elusive. Advances in immunomodulatory therapies have improved disease control for many patients, but relapse rates remain significant, and the cumulative burden of cardiovascular disease continues to drive long-term morbidity and mortality. The ongoing reliance on low-quality evidence and the paucity of RCTs (there remain only 4 RCTs in TAK to date) underscore the urgent need for rigorous, collaborative research efforts. Future research priorities include standardized trial designs, more comprehensive cardiovascular risk screening methods, and international registries. Primarily, future therapeutic strategies must move beyond merely suppressing inflammation to address the dual challenge of immune-mediated vascular injury and progressive cardiovascular risk. Ultimately, the goal must evolve toward a dual mandate: achieving sustained immunological remission while decisively reducing cardiovascular mortality. Collaborative multicenter trials, incorporating cardiovascular end points and supported by international pooled data, will be essential to transform TAK care from its current empiric foundations into a more predictive, personalized, and preventive paradigm.

## Article Information

### Sources of Funding

A. Armstrong is funded by a PhD Studentship from the Kennedy Trust. D. Pugh is funded by a Clinical Lectureship from the Chief Scientist Office (PCL/24/06).

### Disclosures

None.
